# Reproducibility of lymphovascular space invasion (LVSI) assessment in endometrial cancer

**DOI:** 10.1111/his.13871

**Published:** 2019-06-10

**Authors:** Elke E M Peters, Carla Bartosch, W Glenn McCluggage, Catherine Genestie, Sigurd F Lax, Remi Nout, Jan Oosting, Naveena Singh, Huub C S H Smit, Vincent T H B M Smit, Koen K Van de Vijver, Tjalling Bosse

**Affiliations:** ^1^ Department of Pathology Leiden University Medical Center Leiden the Netherlands; ^2^ Department of Pathology Haaglanden Medical Center The Hague the Netherlands; ^3^ Department of Pathology Portuguese Oncology Institute‐Porto Porto Portugal; ^4^ Department of Pathology Belfast Health and Social Care Trust Belfast Northern Ireland UK; ^5^ Department of BioPathology University Paris‐Saclay, Gustave‐Roussy Cancer Center Villejuif France; ^6^ Department of Pathology Hospital Graz II and Medical University of Graz Graz Austria; ^7^ Department of Radiation Oncology Leiden University Medical Center Leiden the Netherlands; ^8^ Department of Cellular Pathology Barts Health NHS Trust London UK; ^9^ Department of Pathological Anatomy Ghent University Hospital Ghent Belgium

**Keywords:** endometrial neoplasms, interobserver study, LVSI, LVSI grading, lymphovascular space invasion, observer variation

## Abstract

**Aims:**

Lymphovascular space invasion (LVSI) in endometrial cancer (EC) is an important prognostic variable impacting on a patient's individual recurrence risk and adjuvant treatment recommendations. Recent work has shown that grading the extent of LVSI further improves its prognostic strength in patients with stage I endometrioid EC. Despite this, there is little information on the reproducibility of LVSI assessment in EC. Therefore, we designed a study to evaluate interobserver agreement in discriminating true LVSI from LVSI mimics (Phase I) and reproducibility of grading extent of LVSI (Phase II).

**Methods and results:**

Scanned haematoxylin and eosin (H&E) slides of endometrioid EC (EEC) with a predefined possible LVSI focus were hosted on a website and assessed by a panel of six European gynaecological pathologists. In Phase I, 48 H&E slides were included for LVSI assessment and in Phase II, 42 H&E slides for LVSI grading. Each observer was instructed to apply the criteria for LVSI used in daily practice. The degree of agreement was measured using the two‐way absolute agreement average‐measures intraclass correlation coefficient (ICC). Reproducibility of LVSI assessment (ICC = 0.64, *P* < 0.001) and LVSI grading (ICC = 0.62, *P* < 0.001) in EEC was substantial among the observers.

**Conclusions:**

Given the good reproducibility of LVSI, this study further supports the important role of LVSI in decision algorithms for adjuvant treatment.

## Introduction

Classic histopathological parameters are the cornerstone of the current risk‐assessment and guide adjuvant treatment for patients with early‐stage (stages I/II) endometrial carcinoma (EC). Tumour factors included in the risk assessment of early‐stage disease are histological type, tumour grade, cervical stromal involvement, depth of myometrial invasion and lymphovascular space invasion (LVSI). Combinations of these factors stratify early‐stage EC patient into low‐risk (LR), high–intermediate risk (HIR) and high‐risk (HR) for recurrence with differential adjuvant treatment choices.^1^, ^2^, ^3^

Currently, significant advances in our understanding of molecular alterations in EC are reshaping the risk assessment by incorporating molecular features. Novel models in which molecular factors are integrated to further refine the risk assessment are being developed.^4^, ^5^ These integrated approaches still rely on the most relevant histological variables mentioned above. The Achilles heel of those histological variables, however, is the reproducibility among pathologists. One of the strongest prognostic variables in this context is the presence (or absence) of LVSI.

LVSI has gained a prominent position in most of the risk stratification systems for EC.^5^, ^6^, ^7^ Adjuvant radiation treatment for patients with grade 1 or 2 stage I EEC is recommended in the presence of LVSI, independent of the depth of myometrial invasion.^7^ It is interesting that the adjective ‘unequivocal’ is used for LVSI in the most recent ESMO–ESTRO–ESGO (European Society for Medical Oncology–European Society for Radiotherapy and Oncology–European Society of Gynaecological Oncology) clinical guidelines,^7^ as it advises to report LVSI only when there is no other interpretation possible. This immediately evokes the question of ‘how reproducible among pathologists is unequivocal LVSI’. In addition, recent work shows that substantial LVSI in EC may have a stronger prognostic significance than focal LVSI;^8^, ^9^ similar effects are reported for LVSI grading in breast cancer.^10^

A diversity of LVSI definitions can be found in the EC literature, reflecting different ways to approach its assessment. Irrespective of the exact formulation, all these refinements are aimed to help distinguish LVSI from LVSI mimics. The most frequently encountered LVSI mimic is artefactual displacement of tumour within myometrial clefts or large endothelial‐lined vessels. These displacements are probably the result of manipulation of the uterus by an intrauterine balloon during surgery^11^ or an artefact induced by inappropriate grossing of a friable tumour.^12^ Artefactual displacement is more likely to occur in cases with poor fixation or in EC with abundant necrosis. Another frequent artefact that mimics LVSI is stromal retraction around invading tumour glands. Furthermore, ‘emboli’ in vascular spaces are not always clearly composed of viable tumour cells. There may be degenerative changes, and infiltration of inflammatory cells may obscure the presence of tumour cells in these emboli. A specific type of myometrial invasion, referred to as ‘microcystic elongated and fragmented (MELF)‐type invasion’,^13^ may also be confused with LVSI, but importantly is also associated with true LVSI. Additional histological criteria, such as proximity to a venous and arterial vessel^10^ or perivascular lymphocytes, have been proposed to favour true LVSI.^14^

The reported prevalence of LVSI in stage I EC varies widely (3.2–35%), indicating that there may be local differences in how LVSI assessment is conducted and reported;^15^, ^16^ however, interobserver variability studies focusing on LVSI in EC are sparse. Given the significance of LVSI evaluation in risk allocation of EC, and the widely accepted difficulties in LVSI assessment, this study was initiated to examine interobserver agreement on the presence of LVSI and LVSI grading. To our knowledge, this is the first study to assess the reproducibility of the recently proposed grading system for LVSI.

## Materials and methods

In a previous study,^8^ haematoxylin and eosin (H&E) stained slides of EEC from 926 patients derived from the PORTEC 1 and 2 trials^2^, ^17^ were locally re‐reviewed for the presence of LVSI by the study pathologists (E.E.M.P., T.B. and V.T.H.B.M.S.). At review, the presence of LVSI mimics was also noted.

In Phase I, to determine agreement of LVSI assessment, 48 cases were selected by the study pathologists, composed of challenging LVSI mimics (*n* = 29) and cases with convincing true LVSI (*n* = 19). The LVSI mimics were composed of MELF (*n* = 8); retraction artefact (no endothelial lining) (*n* = 10); artefactual tumour displacement (*n* = 5); and LVSI mimics of emboli without tumour cells (*n* = 6). H&E slides were scanned and hosted on a website designed for this purpose. To ensure that all observers evaluated the same focus, they were guided to the predefined, digitally annotated putative LVSI focus. It remained possible for the observers to view the whole section and not just the preselected focus by scrolling through the complete scanned slide. In this phase observers were asked to indicate if the selected focus was true LVSI, using the LVSI definition they used in daily practice. When observers did not consider the marked focus as true LVSI, they were asked to specify what type of LVSI mimic was present (Supporting information, Table S1A). In this phase we also asked the observers to explain their choice. We also asked the observers for the definition of LVSI that they used in everyday practice.

In Phase II, we set out to determine agreement of LVSI grading. For this, a new selection of 42 cases was put together by the study pathologists. All 42 cases were considered positive for true LVSI on re‐review and were graded as either focal (*n* = 20) or substantial LVSI (*n* = 22). Cases were presented to the same group of observers on the same website, asking them first to confirm LVSI and next to grade LVSI‐positive cases as either focal LVSI or substantial LVSI. Focal LVSI was defined semiquantitatively as ‘the presence of a single focus of LVSI around the tumour’. Substantial LVSI was defined as ‘diffuse or multifocal LVSI around the tumour’ (Supporting information, Table S1B).^18^ Free text comments were optional.

Six experienced gynaecological pathologists (observers) were recruited via the European Network for Individualised Treatment of Endometrial Cancer (ENITEC) network. We aimed to include pathologists of different nationalities and from different European institutes in order to assure differing training backgrounds.

### Statistics

Raw data were stored on the website, downloaded and processed prior to analysis. Agreement among observers was measured using the two‐way absolute agreement average‐measures intraclass correlation coefficient (ICC). Due to the lack of a gold standard for true LVSI, this method results in a measure of intraobserver and interobserver variability.^19^ The spss version 23.0 package was used for statistical analyses. An ICC value reflects slight (0–0.19); fair (0.2–0.39); moderate (0.4–0.59; substantial (0.6–0.79); or almost perfect (> 0.8) agreement. Additionally, agreement was qualitatively expressed as: ‘full agreement’ when all observers agreed; ‘partial agreement’ when four or five observers agreed and ‘no agreement’ when three or fewer observers agreed.^20^

## Results

Table 1 lists the LVSI definitions provided by the gynaecological pathologists (observers). These definitions all capture the key element of the consensus definition of LVSI; namely, the presence of tumour cells in a vessel lined by endothelial cells. Some observers also include exclusion criteria or components, such as adherence to the vessel wall and the presence of erythrocytes.

**Table 1:** Definitions of LVSI as used by the observers

Observer	What definition of LVSI do you use in daily practice?
A	Cohesive aggregates of tumour cells located inside a vascular space (defined by the presence of an endothelial lining) and preferentially juxtaposed to the vessel wall
B	Carcinoma cells adherent to vessel wall (with endothelial cells)
C	Definite tumour cells within an endothelial lined channel and no features to suggest artefactual vascular invasion
D	Presence of tumour cells in lymphatics or vessels, which is not caused by artefacts (such as smears, retraction)
E	Tumour cells usually as a group or nest within a space that is covered by endothelial cells and does not contain a significant number of erythrocytes
F	The presence of a tumour embolus within a vessel (capillary or lymphatic), usually well defined, rounding up to the contour of the vessel, may or may not be attached to the inner surface, may include red cells or fibrin; absence of marked autolysis

LVSI, lymphovascular space invasion.

### Phase I: reproducibility of LVSI assessment

Full agreement concerning the presence or absence of LVSI was found in 10 of 48 cases (21%); partial agreement in 23 cases (48%); and no agreement in 15 cases (31%) (Table 2). Individual scores are presented in Supporting information, Table S2. One observer was a noted outlier and appeared to have a low threshold for diagnosing true LVSI. Overall, these outcomes resulted in substantial agreement (ICC = 0.6, *P* < 0.001) in LVSI assessment.

**Table 2:** Qualitative level of agreement in LVSI assessment (Phase I) and LVSI grading (Phase II), according to initial central review

Level of agreement	Phase I		Phase II	
	Initial review LVSI‐positive (*n* = 19)	Initial review LVSI‐negative (*n* = 29)	Initial review focal LVSI (*n* = 20)	Initial review substantial LVSI (*n* = 22)
Full	5	5	3	3
Partial	10	13	11	13
None	4	11	6	6

LVSI, lymphovascular space invasion.

**Figure 1:**
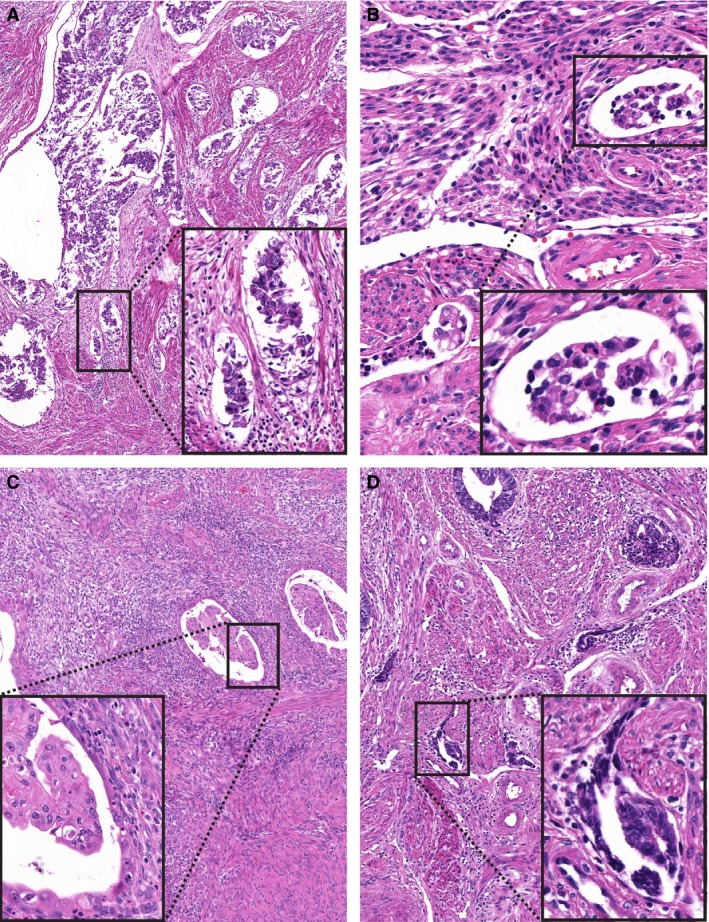
Representative examples of lymphovascular space invasion (LVSI) mimics presented in Phase I. **A**, Retraction artefact around poorly preserved invading tumour. **B**, A cluster of inflammatory cells within a vessel, mimicking tumour cells. **C**, A microcyst aligned by flattened epithelial cells with a cluster of tumour cells in the centre, mimicking true LVSI. **D**, A cluster of tumour cells trapped within a myometrial cleft without an endothelial lining. Note the lack of perivascular infiltrate in all LVSI mimics.

**Figure 2:**
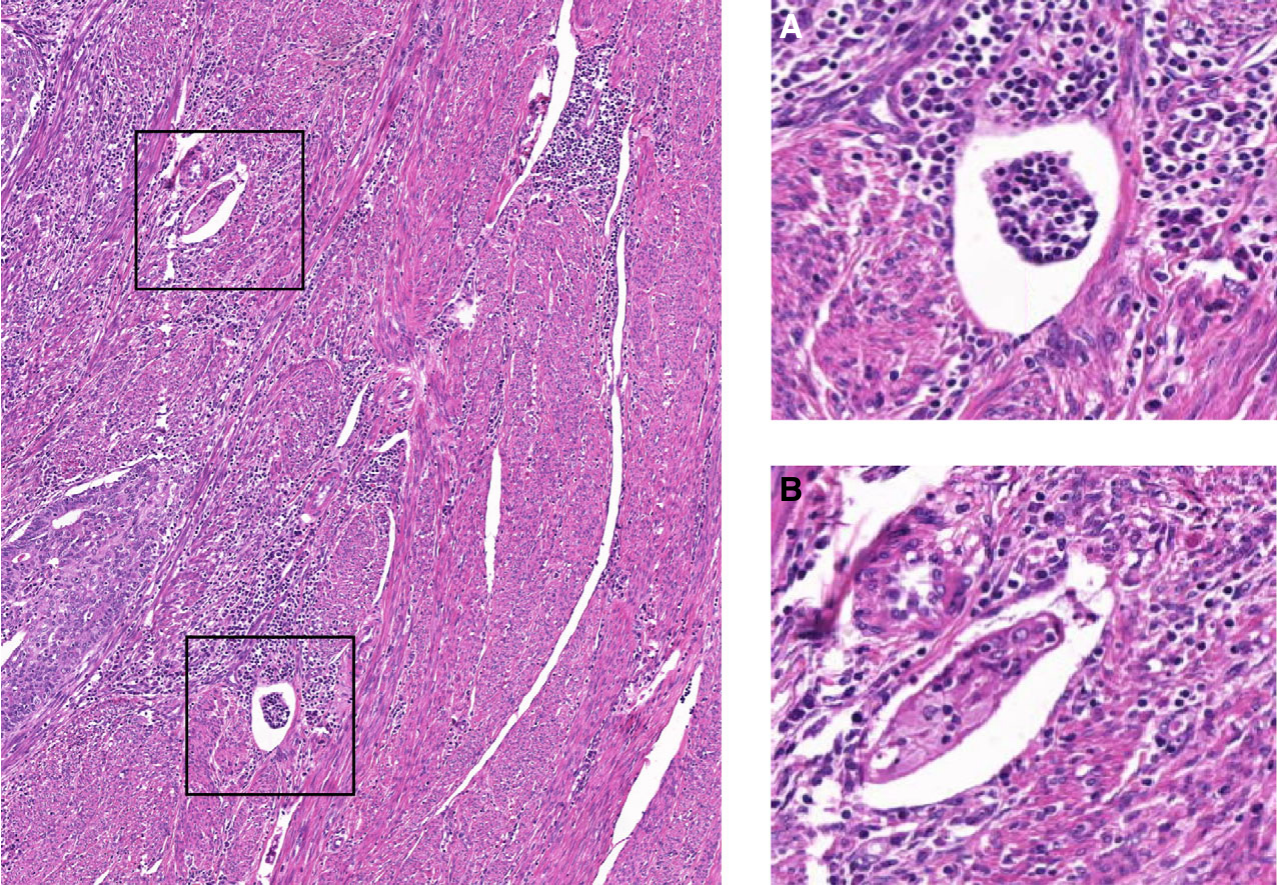
A representative example of a case with no consensus on lymphovascular space invasion (LVSI) assessment. This case shows two suspected foci of LVSI close to each other. The lower focus (**A**) shows the presence of endothelial cells indicating that this is a vessel; however, the cell cluster within this vessel does not unequivocally contain tumour cells. The upper focus (**B**) shows a vessel with a cluster of epithelioid cells infiltrated by a few lymphocytes. Three observers scored this case as LVSI‐positive, two scored negative, arguing the lack of tumour cells, one scored negative because of the lack of endothelial cells. In this case subsequent immunohistochemistry (IHC) would probably result in a higher level of agreement.

Some representative examples of LVSI mimics from the study are illustrated in Figure 1. Interestingly, there was little agreement on the various reasons to score the focus as negative for LVSI. There were 26 cases in which at least two observers stated there was no LVSI. In just eight of these cases (31%) the same explanation was given. In the remaining 18 cases (69%) at least two different reasons for ‘no LVSI’ were given. This is illustrated in Figure 2, a case in which mimics co‐exist resulting in more than one reason to reject true LVSI.

### Phase II: reproducibility of LVSI grading

**Figure 3:**
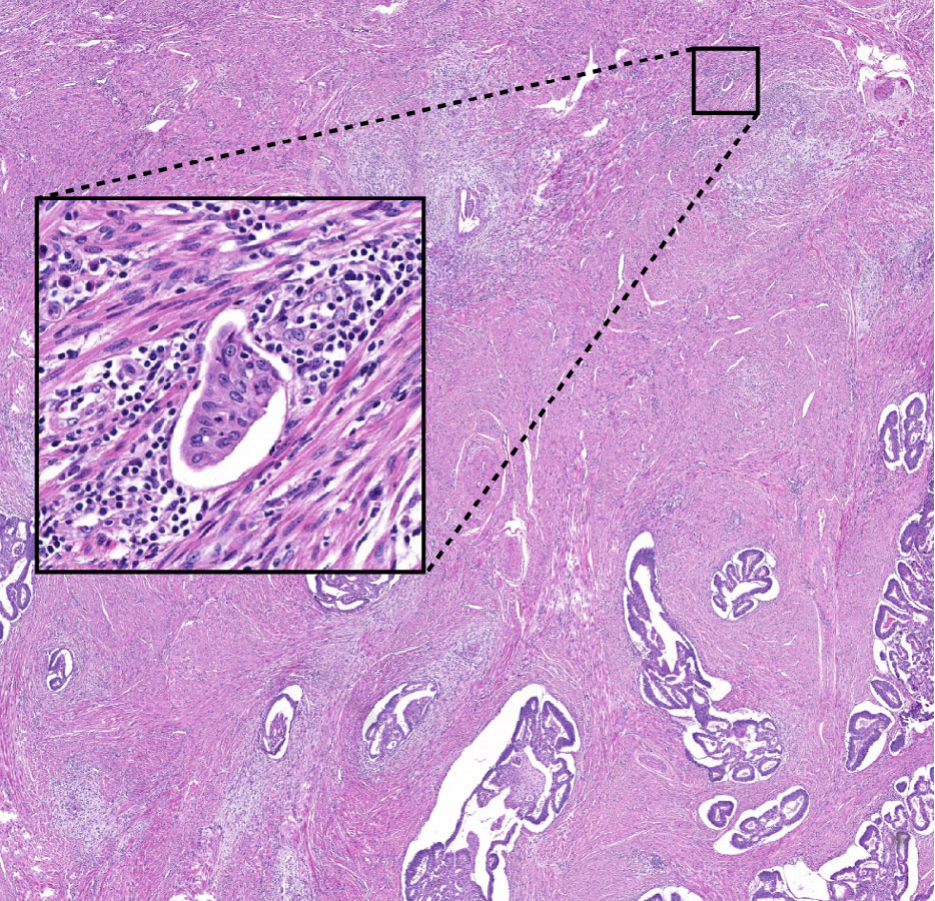
A case derived from Phase II with full agreement on focal lymphovascular space invasion (LVSI). The overview shows infiltrating tumour glands surrounded by an extensive stromal reaction. Some glands are surrounded by retraction artefacts. There is a focus top right (detail shown left) suspected for LVSI. The focus contains a perivascular lymphocytic infiltrate and is adjacent to a venule. This was the only LVSI focus on this haematoxylin and eosin (H&E). All observers graded this as focal LVSI. [Colour figure can be viewed at http://www.wileyonlinelibrary.com]

**Figure 4:**
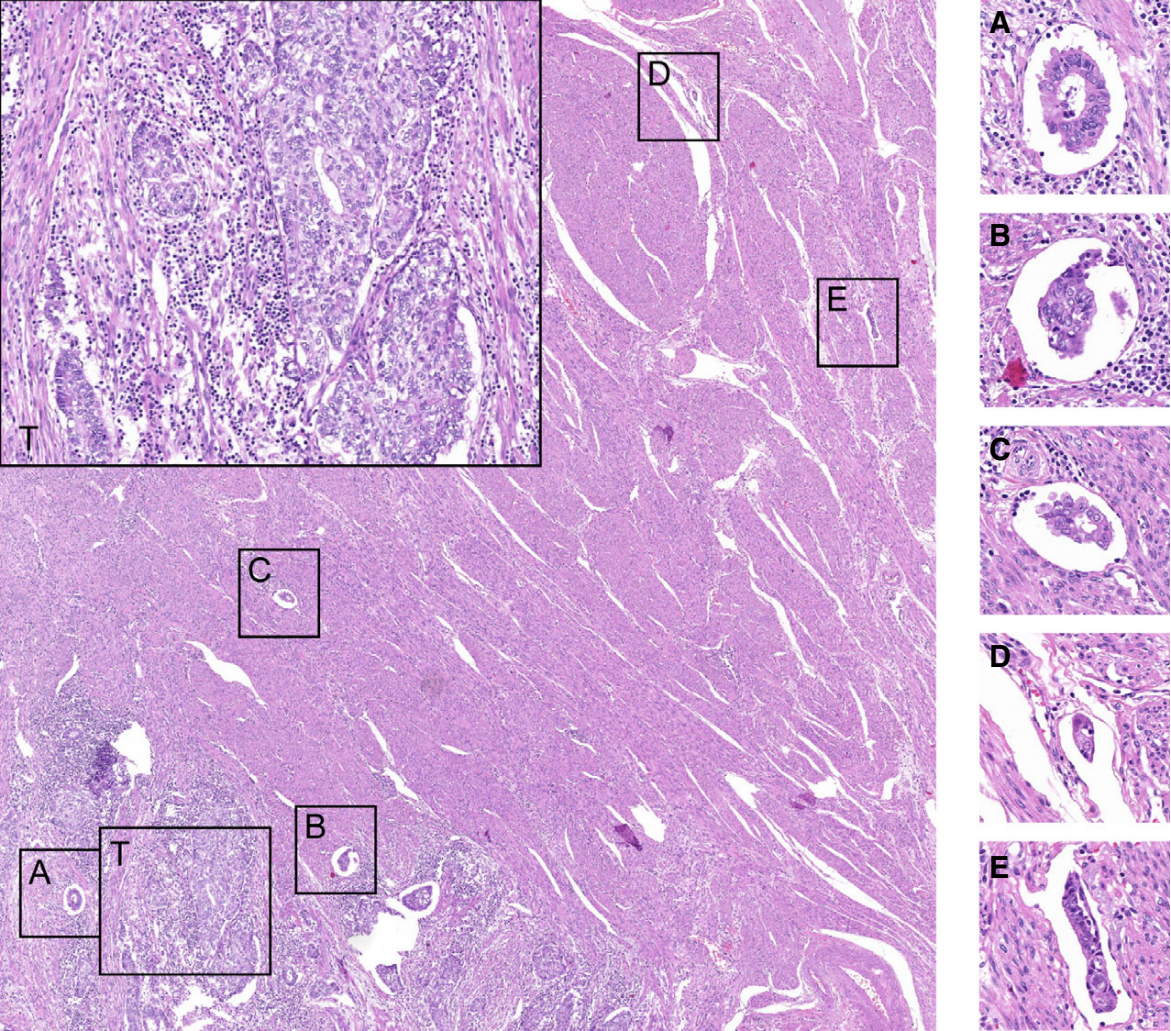
A case derived from Phase II with partial agreement on substantial lymphovascular space invasion (LVSI). Box T shows a detail of the endometrioid endometrial cancer (EEC) with a prominent peritumoral infiltrate. Insets **A–E** show details of putative LVSI foci that were annotated for this case, that was called substantial LVSI by the study pathologist. Five observers diagnosed this case as positive for LVSI and four agreed to grade this as substantial LVSI. [Colour figure can be viewed at http://www.wileyonlinelibrary.com]

Full agreement was achieved in six cases (14%); partial agreement in 23 cases (55%); and no agreement in 13 cases (31%) (Table 2). Figure 3 is an example of a case with full agreement on focal LVSI. Figure 4 illustrates a case with partial agreement on substantial LVSI. The overall reproducibility in this phase was moderate (ICC = 0.54, *P* < 0.001). However, one pathologist consistently scored cases as negative for LVSI, whereas two pathologists had a noted tendency to diagnose substantial LVSI. Individual scores are presented in Supporting information, Table S3. LVSI grading in cases recognised by the observers as true LVSI resulted in substantial agreement (ICC = 0.62, *P* < 0.001) using the predefined semiquantitative definitions for grading LVSI.

## Discussion

In this study we explored the interobserver reproducibility in both diagnosing LVSI and in the application of a recently introduced LVSI grading system.^8^ As the presence of LVSI is considered one of the strongest predictors of recurrence in early‐stage EC, it is critical to assess reproducibility and identify problematic areas to further improve LVSI assessment. Here, we show that gynaecological pathologists reach substantial agreement in LVSI assessment.

We did not provide the observers with a LVSI definition, because a consensus definition for LVSI in the literature is lacking. A variety of elements in the definition of LVSI can be found in the literature, such as the presence of an endothelial lining,^21^ use of ancillary studies,^22^, ^23^, ^24^ position of the LVSI focus relative to the tumour,^25^ attachment of the embolus to the vessel wall or not,^26^, ^27^ the nature of the vessel (lymphatic, vascular, ‘capillary‐like’),^25^, ^28^, ^29^ vitality and shape of the embolus^30^ and presence of surrounding erythrocytes^31^ or perivascular infiltrates.^14^ We did, however, ask our observers to provide the LVSI definition they use in their daily practice. These definitions showed significant overlap, and all LVSI to be defined as ‘tumour cells’ located in a ‘vessel’. The minor differences in refinements to this definition are unlikely to be a source of varying interpretations.

With this study, we add to previous studies regarding reproducibility of pathological reporting of other EC specific characteristics such as histological typing, tumour grading, assessment of cervical involvement and assessment of myometrial invasion.^32^, ^33^, ^34^, ^35^ Levels of reproducibility of these tumour characteristics are similar to our results for LVSI assessment. None of the previous studies specifically focused on LVSI assessment, but there are two studies that report on reproducibility of LVSI in EC.^33^, ^36^ LVSI and other tumour characteristics were reviewed as part of an upfront pathology review before randomisation in the PORTEC‐3 trial.^36^ A high rate of interobserver agreement between the original pathology report and central pathology review was found for LVSI (κ = 0.72). In the study by Guan *et al*., LVSI assessment was part of an alternative binary grading system in EC.^33^ Here, LVSI was defined as clusters of malignant epithelial cells within vascular spaces located outside the main tumour. Assessment was performed on H&E slides and CD31 was used to identify the endothelial lining in indeterminate or suspicious cases. Assessment of 254 EC by four pathologists resulted in a disappointing κ‐value of 0.23 for LVSI. Several explanations may be considered as to why our study resulted in much higher κ‐values. First, LVSI was one parameter among three others, making observers less focused on one particular parameter. Secondly, in our study observers were guided to a predefined focus, ensuring that all observers examined the same area of interest. Lastly, the observers in our study were selected based on their special interest in gynaecological pathology, with the assumption that they are familiar with common LVSI mimics in EC.

Some of the observers in our study commented that they would have used immunohistochemistry (IHC) to prove the presence of endothelial cells in a subset of the presented cases. Although the role of adding IHC to LVSI assessment was not part of the study design, it seems obvious that difficult cases may benefit from the use of IHC. Appropriate IHC to help demonstrate LVSI are pan‐endothelial (CD31) or lymph vessel‐specific (podoplanin/D2‐40) antibodies. Weber *et al*. found that D2‐40 IHC increases the proportion of LVSI‐positive cases in EC compared to H&E evaluation alone. Interestingly, all D2‐40‐positive cases could be identified retrospectively on H&E.^37^ Alexandre‐Sefre *et al*. compared routine H&E LVSI detection with dual pancytokeratin and CD31 staining, and found a threefold increase in the LVSI detection rate from 18% with H&E to 54% using IHC in stage I EC.^24^ However, both studies failed to illustrate how the increased detection with IHC would affect the clinical relevance/prognostic strength of LVSI detection. There may also be reasons to be reluctant to apply IHC universally. Cancer‐associated fibroblasts surrounding adenocarcinoma of the lung^38^ and breast^39^ have been shown to express podoplanin. Although non‐specific fibroblastic reactivity was not described in the studies by Weber *et al*. and Alexandre‐Sefre *et al*., it is possible that an extensive fibroblastic reaction in EC (e.g. in the MELF‐infiltrative growth pattern) could exhibit podoplanin positivity and results in an incorrect diagnosis of LVSI. Furthermore, Harris *et al*, showed that the assessment of both small‐ and large‐vessel involvement in colorectal carcinoma could not be improved by application of D2‐40 and CD31.^40^ We acknowledge, however, that the use of IHC can be useful in selected difficult cases (e.g. cases with extensive retraction artefact), and when used in the correct context will probably further improve interobserver agreement.

Reproducibility of LVSI assessment has also been studied in the context of other tumours, such as hepatocellular carcinoma (HCC),^41^ colorectal cancer^40^ and squamous cell carcinoma of the floor of the mouth.^42^ In the HCC study,^41^ inter‐ and intraobserver reproducibility of six pathologists were analysed. LVSI definitions were not provided and 126 slides and 26 images were circulated twice among the observers. There was moderate overall agreement in both attempts (first round κ = 0.50, second round κ = 0.43), with slightly lower agreement among non‐hepatopathologists compared to hepatopathologists. A study in colorectal cancer^40^ included 50 cases from which one H&E slide circulated among six gastrointestinal pathologists assessing small‐ and large‐vessel invasion using the individual pathologists’ own criteria. The agreement for small‐vessel invasion on H&E slides was fair (κ = 0.28). Agreement was not improved with the use of CD31 (κ = 0.26) or D2‐40 (κ = 0.32). LVSI assessment in squamous carcinoma of the floor of the mouth^42^ was performed on H&E slides from 58 cases by three pathologists using their own criteria. This resulted in substantial agreement for LVSI (κ = 0.64), comparable to our findings. The variation in levels of agreement between these studies shows that reproducibility of LVSI assessment is probably tumour type‐specific.

A three‐tiered LVSI grading system for EEC (no, focal, substantial) has only recently been proposed.^8^ Despite its novelty, this study showed that the observers were able to apply the semiquantitative system with good agreement. Focal LVSI was defined as ‘a single focus of LVSI around a tumour’ and substantial LVSI was defined as ‘diffuse or multifocal LVSI around a tumour’. Given the considerable reproducibility of this system, this seems a very reasonable approach in daily practice. We do, however, recognise that problematic cases exist in which this semiquantitative approach may not suffice. For example, cases with two to five involved vessels, clustered in a small focus, may be regarded as ‘focal’ by some (if assumed that all the foci of LVSI involve a single vessel) and ‘substantial’ by others. Although this scenario is rare and therefore will be a minor problem in practice, the grading system may benefit from more precise cut‐off values. One would anticipate that this would result in further improvement of the reproducibility. At the time of this study, no evidence‐based cut‐off values were available.

Like all interobserver studies, this study is not without its limitations. Importantly, given the lack of a gold standard, we had to rely on the assessment of the study pathologists for case selection. The study cohort was enriched for cases with potential LVSI, including a selection of LVSI artefacts and mimics, and therefore represents a selected and diagnostically difficult cohort. The level of interobserver agreement in this study, therefore, probably represents an underestimation of the true agreement for LVSI assessment in EC. A more realistic unselected routine cohort would include many LVSI‐negative cases without artefacts or mimics, which would probably result in a much higher agreement. Furthermore, we did not provide serial sections or additional stains to the observers, which in selected cases may have improved agreement.

In summary, this study shows that gynaecological pathologists are able to adequately discriminate unequivocal LVSI from LVSI mimics. LVSI grading using a recently proposed three‐tiered system (no, focal, substantial) was reproducible. Given the prognostic relevance,^8^ this study further supports the implementation of this LVSI grading system to routine clinical practice.

## Conflicts of interest

Authors declare there are no sources of support from pharmaceutical or industry companies.

## Supporting information


**Table S1**. (A) Questions and response options in phase 1. (B) Questions and response options in phase 2.
**Table S2**. Raw data phase 1.
**Table S3**. Raw data phase 2.
