# Supplementary material for: Reproducibility of lymphovascular space invasion (LVSI) assessment in endometrial cancer
Source: Histopathology. 2019 Jun 10;75(1):128–36. doi: 10.1111/his.13871 (PMC6852322; doi:10.1111/his.13871)
Supplement: Supplementary file 1 — Table S1. (A) Questions and response options in phase 1. (B) Questions and response options in phase 2. Table S2. Raw data phase 1. Table S3. Raw data phase 2. [file HIS-75-128-s001.docx]

| Take a look at the indicated focus. Do you think this is a focus of LVSI? | |
| --- | --- |
|  | Yes |
|  | No, this is a shrinkage artifact |
|  | No, there is no endothelial lining |
|  | No, there are no tumour cells |
|  | No, this is a focus with MELF pattern invasion (MELF = micro cystic, elongated and fragmented) |
|  | No, this is tumour spill |
|  | No, because of other reasons |
|  | Unsure (explain below) |
| We would like to learn from your answer. You can elucidate your answer in the box below. (optional) | |

Supplementary table 1A. Questions and response options in phase 1.

| Take a look at the indicated focus and its surroundings. Do you think this case shows LVSI? | |
| --- | --- |
|  | No, there is no LVSI |
|  | Yes, this case shows mild LVSI  Definition: mild LVSI = a single focus of LVSI around a tumour. |
|  | Yes, this case shows substantial LVSI  Definition: diffuse or multifocal LVSI around a tumour. |
| We would like to learn from your answer. You can elucidate your answer in the box below. (optional) | |

Supplementary table 1B. Questions and response options in phase 2.

|  | OBSERVER | | | | | |
| --- | --- | --- | --- | --- | --- | --- |
| CASE | A | B | C | D | E | F |
| 1 | 1 | 1 | 1 | 1 | 1 | 1 |
| 2 | 6 | 1 | 6 | 9 | 1 | 6 |
| 3 | 7 | 3 | 1 | 1 | 1 | 1 |
| 4 | 6 | 1 | 6 | 1 | 7 | 6 |
| 5 | 6 | 1 | 6 | 6 | 1 | 1 |
| 6 | 1 | 1 | 1 | 1 | 1 | 1 |
| 7 | 6 | 1 | 1 | 1 | 1 | 1 |
| 8 | 1 | 4 | 4 | 1 | 1 | 3 |
| 9 | 1 | 1 | 1 | 1 | 1 | 1 |
| 10 | 1 | 1 | 3 | 1 | 1 | 1 |
| 11 | 1 | 6 | 9 | 9 | 1 | 1 |
| 12 | 1 | 1 | 1 | 1 | 1 | 9 |
| 13 | 1 | 1 | 1 | 1 | 1 | 1 |
| 14 | 3 | 3 | 9 | 2 | 3 | 2 |
| 15 | 6 | 1 | 1 | 1 | 1 | 1 |
| 16 | 1 | 3 | 5 | 1 | 1 | 1 |
| 17 | 2 | 1 | 1 | 1 | 1 | 3 |
| 18 | 6 | 6 | 7 | 6 | 6 | 6 |
| 19 | 6 | 1 | 9 | 1 | 1 | 1 |
| 20 | 6 | 2 | 1 | 1 | 1 | 1 |
| 21 | 1 | 1 | 1 | 1 | 1 | 1 |
| 22 | 1 | 1 | 9 | 1 | 1 | 1 |
| 23 | 9 | 1 | 1 | 2 | 1 | 2 |
| 24 | 6 | 5 | 1 | 6 | 1 | 1 |
| 25 | 5 | 1 | 1 | 5 | 1 | 5 |
| 26 | 6 | 1 | 1 | 1 | 1 | 9 |
| 27 | 1 | 1 | 1 | 1 | 1 | 1 |
| 28 | 5 | 3 | 5 | 5 | 5 | 5 |
| 29 | 6 | 2 | 6 | 1 | 1 | 1 |
| 30 | 1 | 6 | 6 | 1 | 1 | 7 |
| 31 | 7 | 4 | 1 | 4 | 1 | 1 |
| 32 | 1 | 1 | 1 | 1 | 1 | 1 |
| 33 | 2 | 2 | 1 | 2 | 1 | 2 |
| 34 | 5 | 3 | 1 | 5 | 1 | 5 |
| 35 | 1 | 1 | 5 | 5 | 1 | 5 |
| 36 | 2 | 2 | 1 | 1 | 1 | 2 |
| 37 | 1 | 1 | 6 | 1 | 1 | 1 |
| 38 | 1 | 1 | 9 | 1 | 1 | 1 |
| 39 | 1 | 1 | 1 | 5 | 1 | 2 |
| 40 | 6 | 1 | 1 | 9 | 1 | 9 |
| 41 | 1 | 1 | 1 | 1 | 1 | 2 |
| 42 | 1 | 2 | 1 | 1 | 1 | 1 |
| 43 | 9 | 5 | 1 | 6 | 1 | 2 |
| 44 | 1 | 2 | 1 | 1 | 1 | 1 |
| 45 | 4 | 4 | 4 | 4 | 1 | 6 |
| 46 | 3 | 3 | 1 | 3 | 1 | 2 |
| 47 | 4 | 4 | 9 | 1 | 1 | 1 |
| 48 | 1 | 1 | 1 | 1 | 1 | 1 |

Supplementary table 2. Raw data phase 1. Key: A to F: observers. 1: LVSI positive; 2: No LVSI – shrinkage; 3: No LVSI – no endothelial lining; 4: No LVSI – no tumour cells; 5: No LVSI – MELF; 6: No LVSI – spill; 7: No LVSI – other reasons; 9: Unsure.

|  | OBSERVER | | | | | |
| --- | --- | --- | --- | --- | --- | --- |
| CASE | A | B | C | D | E | F |
| 1 | 2 | 2 | 2 | 2 | 2 | 2 |
| 2 | 1 | 1 | 2 | 0 | 2 | 2 |
| 3 | 1 | 2 | 1 | 2 | 2 | 2 |
| 4 | 1 | 2 | 2 | 2 | 2 | 2 |
| 5 | 0 | 1 | 2 | 1 | 2 | 2 |
| 6 | 2 | 1 | 2 | 2 | 1 | 2 |
| 7 | 2 | 1 | 2 | 2 | 1 | 2 |
| 8 | 1 | 0 | 2 | 1 | 1 | 0 |
| 9 | 0 | 2 | 2 | 2 | 2 | 2 |
| 10 | 2 | 2 | 2 | 2 | 2 | 2 |
| 11 | 2 | 2 | 0 | 2 | 2 | 2 |
| 12 | 0 | 0 | 2 | 2 | 1 | 1 |
| 13 | 1 | 1 | 1 | 1 | 1 | 1 |
| 14 | 0 | 1 | 2 | 1 | 2 | 2 |
| 15 | 0 | 1 | 1 | 0 | 1 | 1 |
| 16 | 0 | 1 | 2 | 1 | 2 | 2 |
| 17 | 1 | 1 | 2 | 2 | 2 | 2 |
| 18 | 2 | 2 | 2 | 1 | 2 | 2 |
| 19 | 1 | 1 | 2 | 2 | 2 | 2 |
| 20 | 1 | 1 | 0 | 0 | 1 | 1 |
| 21 | 2 | 2 | 2 | 2 | 2 | 2 |
| 22 | 0 | 2 | 2 | 2 | 2 | 2 |
| 23 | 0 | 0 | 2 | 1 | 1 | 1 |
| 24 | 2 | 1 | 2 | 2 | 2 | 2 |
| 25 | 2 | 2 | 1 | 2 | 2 | 2 |
| 26 | 2 | 0 | 2 | 1 | 2 | 2 |
| 27 | 1 | 1 | 2 | 2 | 1 | 2 |
| 28 | 2 | 1 | 2 | 2 | 2 | 2 |
| 29 | 0 | 1 | 2 | 2 | 2 | 2 |
| 30 | 0 | 2 | 2 | 2 | 2 | 2 |
| 31 | 2 | 1 | 2 | 0 | 1 | 2 |
| 32 | 1 | 2 | 2 | 2 | 2 | 2 |
| 33 | 1 | 1 | 2 | 1 | 2 | 2 |
| 34 | 0 | 2 | 2 | 2 | 1 | 2 |
| 35 | 2 | 2 | 2 | 2 | 2 | 2 |
| 36 | 2 | 2 | 2 | 2 | 2 | 2 |
| 37 | 2 | 1 | 2 | 1 | 1 | 2 |
| 38 | 1 | 0 | 2 | 0 | 1 | 2 |
| 39 | 1 | 1 | 2 | 0 | 2 | 2 |
| 40 | 0 | 1 | 2 | 1 | 1 | 2 |
| 41 | 0 | 2 | 2 | 2 | 2 | 2 |
| 42 | 0 | 2 | 2 | 0 | 2 | 2 |

Supplementary table 3. Raw data phase 2. Key: A to F: observers. 0: no LVSI; 1: focal LVSI; 2 substantial LVSI.
